# Synthesis of Tubular Hydroxyapatite and Its Application in Polycaprolactone Scaffold Materials

**DOI:** 10.3390/jfb15010022

**Published:** 2024-01-14

**Authors:** Ziyi Hong, Shaohui Wang, Fengyu Liu

**Affiliations:** Department for Materials Science and Engineering, East China Jiao Tong University, Nanchang 330013, China; 2021151002000123@ecjtu.edu.cn (Z.H.); 2021151002000324@ecjtu.edu.cn (F.L.)

**Keywords:** PCL, tubular HAp, artificial bone scaffold, γ-aminopropyltriethoxysilane (KH550), solvothermal reaction method

## Abstract

Nano-hydroxyapatite (HAp) is an ideal material in the field of biomedicine due to its good biocompatibility and bioactivity. However, a significant drawback of pure HAp materials is their inferior mechanical properties. Therefore, in this rigorous investigation, the optimal calcium-to-phosphorus ratio for the synthesis of HAp was meticulously delineated, followed by its nuanced modification using KH550 (γ-aminopropyltriethoxysilane). This was further amalgamated with polycaprolactone (PCL) with the aim of providing a superior material alternative within the domain of bone scaffold materials. The post-modified HAp demonstrated enhanced interfacial compatibility with PCL, bestowing the composite with superior mechanical characteristics, notably a peak bending strength of 6.38 ± 0.037 MPa and a tensile strength of 3.71 ± 0.040 MPa. Scanning electron microscope (SEM) imagery revealed an intriguing characteristic of the composite: an initial ascension in porosity upon HAp integration, subsequently followed by a decline. Beyond this, the composite not only exhibited stellar auto-degradation prowess but also realized a sustained release cycle of 24 h, markedly optimizing drug utility efficiency. A kinetic model for drug dispensation was developed, positing an adherence to a pseudo-second-order kinetic principle. In tandem, through the formulation of an intra-particle diffusion model, the diffusion mechanisms pre- and post-modification were deeply probed. Cytotoxicity assays underscored the composite’s exemplary biocompatibility. Such findings accentuate the vast potential of the modified HAp–PCL composite in bone tissue engineering, heralding a novel and efficacious avenue for impending bone defect amelioration.

## 1. Introduction

Bones, predominantly composed of 70% Nano HAp and 30% collagen, represent the most rigid tissue within an organism [1,2,3]. Integral to the structural architecture of humans, they not only provide essential support, facilitate movement, and shield vital organs but also play pivotal roles in modulating blood pH and ensuring the equilibrium of calcium and phosphate in metabolic activities [4,5]. However, as human lifespans increase and the population ages, the number of individuals with bone diseases has risen significantly [6]. Additionally, improper protection during exercise could cause damage to bones and joints, further increasing the need for bone therapy. Biological scaffold materials, widely used in load-bearing applications like orthopedic implants, bifurcate into two predominant categories: the permanent kind for comprehensive joint replacements, including the hip, knee, shoulder, elbow, and ankle; and the temporary kind, featuring elements such as screws, plates, wires, pins, rods, and intramedullary nails [6,7,8]. Currently, the predominant biological scaffold materials in clinical settings are primarily metallic. These metal scaffolds, while prevalent, present several clinical challenges, including suboptimal biocompatibility, a propensity for secondary fractures, and the deleterious release of metal ions detrimental for human health [9,10,11]. Moreover, the scaffold materials implanted into the body need a secondary surgery to be removed [12]. During this time, it would inevitably cause many inconveniences to patients’ lives. The release of toxic metal ions may even endanger the life and health of patients. In 1993, Professor Langer and Professor Vacanti first proposed the concept of tissue engineering in the journal Science (Langer R, Vacanti J, 1993) [13]. It has continued to develop and grow in the following decades. The value of PCL scaffold materials in the fields of medicine and biomedical engineering is gradually gaining recognition [14,15]. PCL is a biodegradable polyester, commonly used in the fabrication of scaffolds, which serve as temporary structures in tissue engineering. These scaffolds can assist in the growth of cells, forming the desired tissue structures [16]. However, as a polymer, pure PCL exhibits considerable toughness but lacks sufficient strength [17]. Simultaneously, Nadeem Siddiqui noted in their review that the hydrophobic nature of pure PCL scaffold materials is adverse to cell attachment and proliferation [18]. Therefore, the fabrication of performance-enhanced composite materials through the combination of PCL with other materials has become a key research direction [19].

HAp is renowned for its biocompatibility and the generation of benign by-products during degradation within the human body, a stark contrast to the widely used clinical materials like stainless steel and titanium alloys. Unlike these metals, HAp does not release harmful ions into the body [20]. It also shields patients from potentially severe pain, medical complications, or the risk of re-fracture, thereby conserving both human and financial resources. Furthermore, the mechanical properties of synthetic bone scaffold materials surpass those of natural human bones [21]. Due to its bone-like chemical composition, HAp is widely used in the field of biomaterials [22]. HAp demonstrates lower toughness in its mechanical properties, and the process of shaping it processes certain difficulties. Toughness enhancement in hydroxyapatite-based composites is necessary [23]. At present, extensive academic research indicates that HAp/PCL composite materials demonstrate significant potential in the field of bone scaffold applications. These materials have been confirmed to possess excellent biocompatibility, yet their mechanical properties still require further enhancement (Milovac, D. et al., 2004) [24,25]. Valarmathi pointed out that the introduction of PCL in the composite can reach the stage that the composites are blood compatible with the hemolytic ratio of less than 2% [26].

However, as HAp is an inorganic hydrophilic material and PCL is an organic hydrophobic polymer, simply mixing them can inevitably lead to a series of problems, such as poor dispersion, interfacial effects, stress concentration, and the formation of micro-voids within the material, which would reduce the material’s strength. Therefore, finding ways to improve the compatibility between HAp and PCL becomes key to solving these issues. Lee pointed out that surface-modified HAp showed a markedly improved dispersion in methylene chloride compared to unmodified HAp. Furthermore, as the quantity of grafted PCL on the HAp increased, its colloidal stability experienced a significant enhancement [27]. Shuai C et al. discussed the modification of HAp using phosphonic acid coupling agents of nanoparticles and their composite with PLLA and discussed the interfacial effect of PLLA/HAp in bone scaffolds [28,29]. The mechanical properties of the modified material have been significantly improved. Kim pointed out that surface-modified silk hydrogel containing nanoparticle HAp with hyaluronic acid–dopamine conjugates could make contributions to the proliferation of cells, and due to its remarkable capability to promote osteoblast cell proliferation, coupled with its highly porous structure, this hydrogel scaffold is well suited for application in bone regeneration [30]. In this study, KH550 was employed as a modifying agent. As a silane coupling agent, KH550 possesses a unique ability to form chemical bonds between inorganic and organic materials, significantly enhancing the compatibility between these two distinct types of materials. By facilitating this form of interfacial bonding, KH550 plays a pivotal role in improving the overall performance of the composite material, enabling a more effective integration of inorganic and organic components [31].

Efforts are being made to improve the mechanical properties of medical-grade HAp to fulfill the requirements of medical material standards [32]. This is challenging due to the limited range of biocompatible elements available and the need to comply with their specific composition thresholds as determined by toxicology guidelines. This investigation delves into the influence of varying Ca/P ratios on the morphology of nano HAp, pinpointing the optimal Ca/P ratio that yields nano HAp powder with an elevated aspect ratio. The amalgamation of these two materials unveils a prominent interfacial effect. Consequently, KH550 was employed to modify the nano HAp, subsequently amalgamating it with PCL resin. Acting as a silane coupling agent, KH550 not only augments the compatibility between HAp and PCL but also bolsters the mechanical prowess of the composite material. After the KH550 modification, the dispersion of nano HAp within the PCL matrix saw marked enhancements. Observations via SEM revealed that a judicious inclusion of modified nano HAp particles resulted in their homogeneous dispersion within the PCL matrix, conspicuously devoid of agglomeration. This equitable distribution aids in amplifying the composite material’s overarching mechanical attributes. Moreover, the composite material manifests a three-dimensional porous structure, fostering the efficient transmission of nutrients and the effective elimination of metabolic wastes from cells and tissues. Its high porosity allows for the embedding of functional drugs and ensures their sustained release within biological entities. Consequently, this paper examines the drug-release mechanisms of the composite material, building a model to fit. It also embarks on an extended tracking experiment on the material’s in vitro degradation. The scaffold, being degradable within the human anatomy, carves out space for bone regeneration, actualizing autologous bone grafts. Employing the CCK8 and live–dead methodologies, the material’s toxicology was scrutinized, confirming the scaffold’s non-cytotoxic nature.

## 2. Experimental

### 2.1. The Preparation of HAp Nanorods

The HAp used for the experiments was prepared using the solvothermal reaction method. CaCl_2_ solution (0.440 g, AR, ShanTou Xilong Chemical Reagent, Shantou, China) and NaOH solution (20 mL, 1.65 M, AR, Xilong Chemical Reagent, Shantou, China) were added to the mixture of ethanol and oleic acid in a certain proportion at each 10-min interval under magnetic stirring (400 rpm at room temperature). (NaHPO_4_)_6_ (20 mL, 0.018–0.04 M) was dropped into the mentioned solution after completing the previous step. At this moment, the total volume of the reaction solution was 100 mL. Then, the reaction-mixed solution was put into a stainless steel autoclave lined with tetrafluoroethylene, heated at 180 °C for 5–35 h in an electric blast drying oven, and then naturally cooled to room temperature.

**Figure 1:**
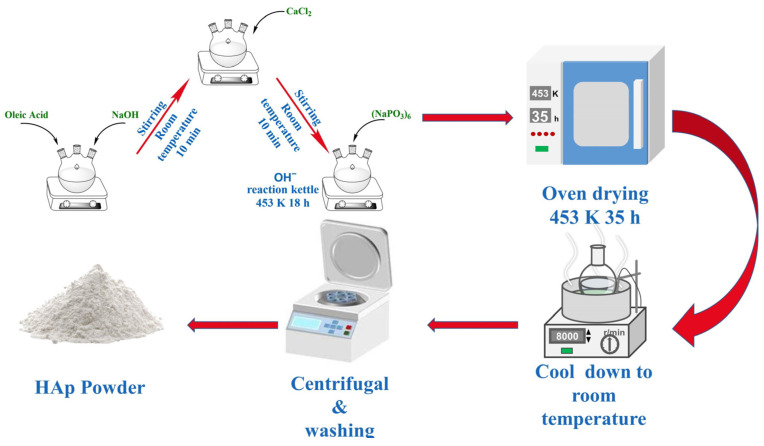
Preparation process of HAp.

Ultimately, the resultant compounds were isolated using centrifugation (KATE Experimental Instrument Co., Ltd., Chengdu, China, TG16B, 8000 rpm). Subsequently, they were purified with three successive rinses in deionized water and ethanol and then desiccated at 50 °C overnight, culminating in the acquisition of a pristine white powder. The above steps are listed in the form of images, as shown in Figure 1. The nano HAp prepared under different calcium-to-phosphorus ratios was analyzed using a transmission electron microscope (TEM, PerkinElmer, Waltham, MA, USA) to select the most suitable calcium-to-phosphorus ratio conditions. Subsequent experiments were conducted with the nano HAp prepared under the above conditions.

### 2.2. The Preparation of KH550-HAp/PCL Composites

KH550, an organosilane coupling agent, is commonly employed to enhance interfacial compatibility between inorganic substances and organic polymers. Upon the chemical interaction between HAp and KH550, the silane coupling agent can establish an organic layer on top of the HAp surface, thereby amplifying the adhesive force between HAp and organic polymers, such as PCL. Such modifications serve to augment the dispersion of HAp within composite materials and refine its interfacial bonding with the foundational polymer matrix. Specifically, the triethoxy group in KH550 reacts with the surface hydroxyl group of HAp, while the γ-amino group can engage with myriad organic substances. This approach proves exceptionally effective for the fabrication of HAp/polymer composites, particularly when HAp is utilized as a filler or a reinforcing agent within biomaterials. KH550 was employed to modify tubular HAp, thereby enhancing its affinity with PCL. In this experiment, the amount of KH550 added constituted 1.5% of the total mass of HAp. The effect of different modification times (0, 1, 7, 18, 24, and 36 h) on the material was discussed in subsequent content.

After completing the modification of HAp, the HAp/PCL nanocomposites were synthesized utilizing the solution blending approach. Initially, PCL, possessing an average molecular weight of 80,000, was dissolved in CH_2_Cl_2_, maintaining a mass ratio of PCL to CH_2_Cl_2_ at 1:2, and the PCL was heated at a constant temperature in a water bath at 60 °C to make it completely dissolved in CH_2_Cl_2_. Subsequently, the HAp suspension was poured into the solution. An ultrasonic machine (Ampu Scientific Instrument Co., Ltd., Shanghai, China) was used to shake the solution for 10 min and then stir it intensively for 24 h at a speed of 350 rpm under mechanical stirring, so that both could be fully mixed. The procured HAp/PCL slurry was introduced to a substantial volume of anhydrous ethanol, and it was subsequently transferred to a mold for shaping, only ensuring its removal post-complete solvent evaporation.

### 2.3. Characterization

The effect of different calcium–phosphorus ratios on the morphology of HAp was observed using a TEM (TEM-2010; PerkinElmer, Waltham, MA, USA). The samples used for TEM characterization were dispersed in absolute ethanol and ultrasonicated before observation. The morphological changes in KH550-modified tubular HAp at different modification times was observed using a SEM (SU8010, Hitachi, Ltd., Tokyo, Japan; 10 kV). The selected area electron diffraction (SAED) was observed using a SEM at 10 kV. The surface morphology of KH550-HAp/PCL composite materials impact section analysis was observed using a SEM (SUS8010; 5 kV). BET surface areas were measured through nitrogen adsorption at 77 K using a Quantachrome NOVA 2200e instrument (Quantachrome, Boynton Beach, FL, USA) The samples were degassed for 12 h, at 95 °C, before the measurements took place in order to eliminate the air and moisture present in the samples. The Brunauer–Emmett–Teller (BET) model was applied to fit the isotherms and calculate the specific surface area of the materials. The components of HAp was analyzed using an Axis Ultra X-ray photoelectron spectroscope (XPS, ESCALABxi+, Waltham, MA, USA) equipped with a standard monochromatic Al-kα source ($h_{v}$ = 1486.6 eV).

### 2.4. Mechanical Properties

Tensile and bending strength tests were carried out under ambient conditions. The mechanical properties of the material with different (0% 2% 4% 8% 10% and 15%) $w_{t}$% of HAp was determined using an electronic universal testing machine. The specific details are listed in Table 1. The tensile strength and bending strength tests followed the ASTM D638 and ASTM D790 standards, respectively [33,34]. The samples for testing were prepared according to the ASTM standards: For tensile strength (ASTM D638), the dimensions were 165 mm in length, 13 mm in width, and 3.2 mm in thickness, with a gauge length of 50 mm. For bending strength (ASTM D790), the dimensions were 127 mm long, 12.7 mm wide, and 3.2 mm thick, as well as a gauge length of 50 mm. Five parallel experiments were conducted, and the average value was taken with the calculation of the standard deviation.

### 2.5. Porosity Test

The total porosity of the material with different (0% 2% 4% 8% 10% and 15%) $w_{t}$% of modified HAp composite was determined using the liquid displacement method. Ethanol was used as a liquid to soak the synthesized composites. Porosity measurements were taken at different time intervals (24 h, 48 h, and 72 h). The composite with different $w_{t}$% of HAp was immersed in a known volume ($V_{1}$) of ethanol. The ethanol (total volume) and the volume after the immersion of the composite were recorded ($V_{2}$). The composite was removed from the ethanol, and then the residual ethanol volume ($V_{3}$) was measured. The same procedure was repeated three times, and the average of the results was taken. The percentage of porosity was calculated by utilizing Equation (1):

(1) $$Porosity\;(\%)=\frac{V_{1}-V_{3}}{V_{2}-V_{3}}$$

### 2.6. Drug In Vitro Drug-Release Studies

The in vitro drug release of ibuprofen from the optimized batch of HAp/KH550-PCL nanoparticles was studied using the dialysis sac method. A dialysis sac containing 4 mL of nanoparticulate formulation (ibuprofen: 4 mg) was tied with a thread and immersed in 100 mL of 1 X PBS buffer (release media). Aliquots of 2-mL samples were taken at regular time intervals, and the volume of the medium was maintained by replacing it with fresh PBS. The ibuprofen contents in the samples were determined through measuring the absorbance at 220 nm.

### 2.7. In Vitro Degradation Tests

After undergoing 4 h of UV light sterilization (800 W; 253.7 nm), the rectangular material samples with a known initial weight ($m_{0}$) were placed into tubes containing 50 mL of PBS (pH = 7.4). These tubes were then incubated at 36.5 °C. The specimens were sampled on days 0, 7, 14, 21, 28, 42, 56, 70, 100, 130, 160, 190, 220, 250, and 300. The sample selected was dried at 50 °C; then, its mass, $m_{t}$, was accurately measured and recorded.

### 2.8. Kinetic Model of Drug Release

In this research, modified HAp and the HAp before modification were selected to explore their adsorption kinetics. The pseudo-first-order kinetic model (Equation (2)), the pseudo-second-order kinetic model (Equation (3)), and the intraparticle diffusion kinetic model (Equation (4)) were established as follows:

(2) $$q_{t}=\frac{q_{e}^{2}k_{2}t}{1+q_{e}k_{2}t}$$

(3) $$q_{t}=k_{p}t^{0.5}+C$$

(4) $$q_{t}=q_{e}\times (1-e^{-k_{1}t})$$

where $k_{1}$ and $k_{2}$ represent the reaction rate constants of the pseudo-first-order and pseudo-second-order kinetic models, respectively. $k_{p}$ represents the diffusion rate constant in the particle, and the adsorption constant C represents the intercept. In addition, the particle internal diffusion model was selected to further explore the diffusion mechanism, and the drug adsorption kinetics of tubular HAp was evaluated together with the pseudo-first-order kinetic model and pseudo-second-order kinetic model.

### 2.9. Cytotoxicity Test

The CCK-8 assay was performed to examine the viability of the cultured MC3T3-E1 cells (shiyanjia lab. Ltd., Nanchang, China), following the manufacturer’s instructions. For the cytotoxicity assay of the HAp/PCL, MC3T3-E1 cells were seeded into 48-well plates at a density of $2\times 10^{4}$ cells per well and cultured for 24 h prior to the assay; then, the medium was replaced with fresh medium containing various amounts of HAp/PCL; their concentrations were 1, 10, 50, 100, and 200 μg/mL. After 24 h of co-cultivation, the medium was replaced with fresh DMEM medium containing 10% CCK-8 working solution and incubated for another 3 h. The optical density of the supernatant was measured and recorded using a microplate reader.

### 2.10. Statistical Analysis

SPSS (version 25.0, IBM, Amonk, NY, USA) and Origin (Origin 2022, OriginLab Corporation, Northampton, MA, USA) software were used for data processing and image drawing. One-way analysis of variance (ANOVA) and Duncan’s multiple comparison method were used to analyze the data. *p* < 0.05 indicates significant differences, while *p* > 0.05 indicates no significant differences between the data [35].

## 3. Results and Discussion

### 3.1. Synthesis of HAp Nanorods

**Figure 2:**
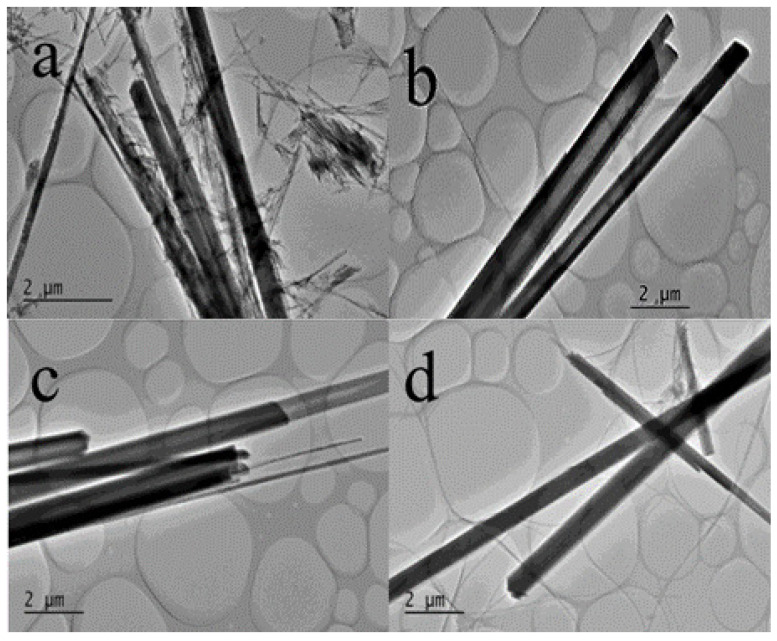
TEM images of HAp obtained for different Ca/P molar ratios ((**a**) Ca/P = 0.7; (**b**) Ca/P = 1.0; (**c**) Ca/P = 1.3; and (**d**) Ca/P = 1.6).

By adjusting the Ca/P molar ratio in the solution before the solvothermal reaction, this study successfully explored the effect of the addition of calcium and phosphorus sources on the morphology of the product. Figure 2a–d present the TEM images of HAp synthesized for different Ca/P molar ratios (a: Ca/P = 0.7; b: Ca/P = 1.0; c: Ca/P = 1.3; and d: Ca/P = 1.6). The diameters of a–d were 604 nm, 1287 nm, 1276 nm, and 846 nm, respectively.

It can be seen from Sample A that synthesized HAp tended to form linear or tubular HAp; however, the formation state is unstable, messy, and extremely unsmooth. Sample B represented the characteristic that HAp basically presents a tubular shape, and its surface is relatively smooth. Sample C represented the characteristic that HAp presents: the coexistence of tubular and linear morphology, and its surface is also relatively smooth; Sample D, under the Ca/P molar ratio of 1.6, represented the characteristic that all HAp presents a linear morphology and a smooth surface. In general, with an increase in the calcium–phosphorus ratio, the morphology of HAp tends to be stable and smooth. At the Ca/P molar ratio of 1.0 (Sample B), HAp forms a tubular structure with a stable distribution and a smooth surface. In subsequent experiments, the HAp used was the one with a Ca/P ratio of 1.0, which shows a stable distribution and a smooth surface.

### 3.2. Infrared Spectroscopy (FT-IR) Analysis of Modified Tubular HAp

**Figure 3:**
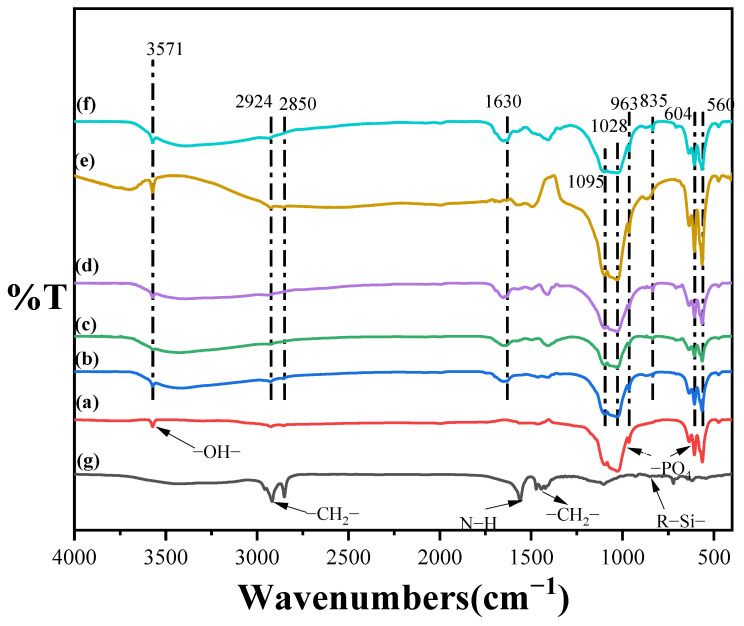
(a–f) The FT-IR spectra of KH550-HAp with different modification times (0, 1, 7, 18, 24, and 36 h). (g) FT-IR image of KH550.

As shown in Figure 3, curves a–f are the FT-IR curves of KH550-HAp at different reaction times (0, 1, 7, 18, 24, and 36 h), and curve g is the FT-IR plot of KH550. In the IR absorption peaks of curves a–f, the symmetric and anti-symmetric stretching vibration peaks of the ${PO}_{4}^{3-}$ groups appeared at 1095 cm^−1^, 1028 cm^−1^ and 963 cm^−1^_,_ respectively, while the characteristic peaks at 606 cm^−1^ and 566 cm^−1^ were attributed to the in-plane bending vibration of the ${PO}_{4}^{3-}$ groups, and the IR characteristic peaks at 3571 cm^−1^ originated from the O-H stretching vibration in HAp. The absorption peaks at 2858 cm^−1^ and 2934 cm^−1^ correspond to the stretching vibration of -C-H- in KH550, while the characteristic absorption peak at 1405 cm^−1^ may be the result of the C-H in-plane bending vibration, the presence of which may originate from the methyl group on KH550 with only a slight shift. The absorption peak at 1630 cm^−1^ may correspond to the C-N stretching vibration on the molecular chain of KH550, and the absorption peak at 835 cm^−1^ should correspond to the stretching vibration of the R-Si-O groups on silane. In conclusion, KH550 grafted with hydroxyl groups on the surface of HAp after hydrolysis to form hydrogen bonds and condensed into -Si-O- groups, proving that KH550 successfully interacts with tubular HAp.

### 3.3. SEM Analysis of Modified Tubular HAp

**Figure 4:**
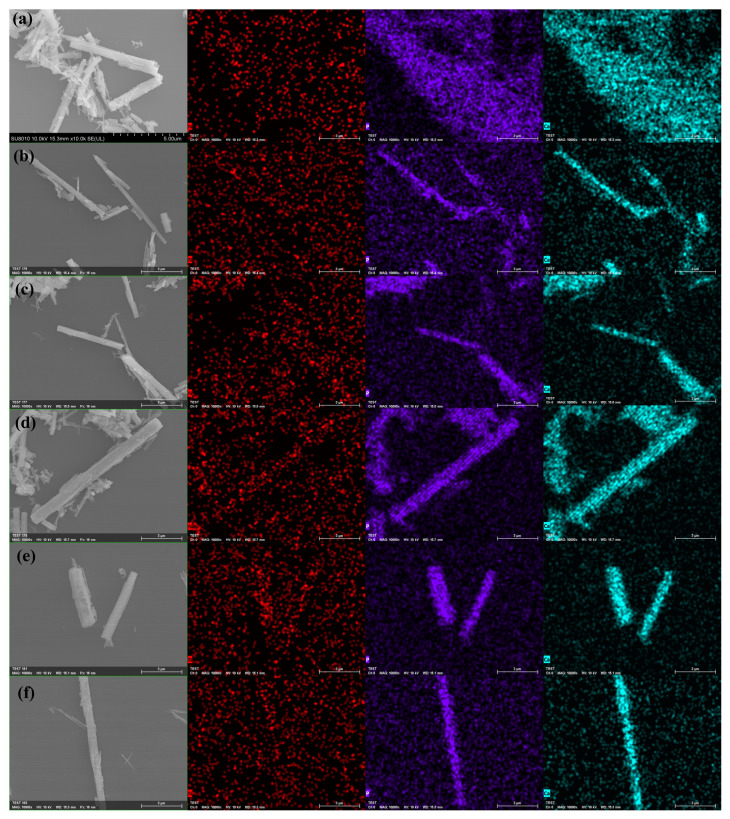
The SEM image of KH550-HAp at different modification times. ((**a**–**f**) represents 0, 1, 7, 18, 24, and 36 h) and the mapping N, P, and Ca images.

To vividly observe the morphological changes in KH550-modified tubular HAp at different modification times, SEM observations were conducted on six groups of samples, and the distribution of key elements was analyzed through SEM and mapping tests. This further substantiated the role of KH550 in modifying HAp. As shown in Figure 4, an increase in surface roughness was observed in samples (a) to (f) following their KH550 modification, likely attributable to the alteration in the HAp surface morphology due to the attachment of KH550. In the corresponding mapping scans, the N element, unique to KH550, was specifically examined. In image (a), the N element appeared to be initially uniformly distributed in the blank areas. As the modification time progressed, the distribution of N shifted, gradually concentrating from the blank parts to the sample areas, revealing the sample contours in the N element observation images. This indicated the successful bonding of KH550 on the HAp surface and its effective modification. The samples in Group E were observed to display the highest density of red dots within their morphological range, which is considered to indicate the maximum degree of grafting of KH550 molecules. The phosphorus element image (purple light spots in the figure) and the calcium element image (cyan light spots in the figure) effectively reflect the morphological characteristics of HAp.

### 3.4. Analysis of the Specific Surface Area (BET) of Modified Tubular HAp

**Figure 5:**
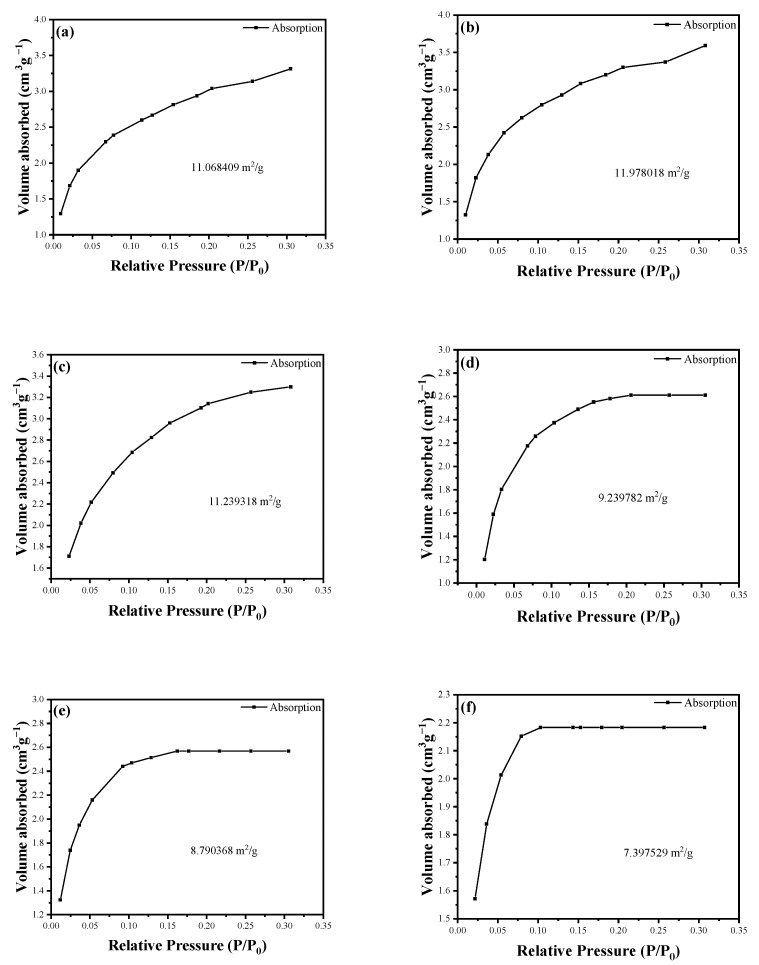
The specific surface area of HAp at different modification times. ((**a**–**f**) represents 0, 1, 7, 18, 24, and 36 h).

To clarify the structural property changes in the sample during the modification process, BET surface area analysis was employed through N_2_ adsorption isotherm measurements. In Figure 5, curves a–f correspond to HAp with the modification times of 0, 1, 7, 18, 24, and 36 h, respectively. Within the first hour of the modification reaction, a slight increase in surface area was observed, reaching 11.9780 m^2^/g. This increase is likely due to the minimal attachment of KH550 to HAp, slightly enhancing the surface area of HAp. However, with the reaction’s progression, especially after 7 h, the surface area was found to decrease to levels comparable to the initial values. This decrease is probably a result of the tubular structure of HAp being internally covered as KH550 enters, affecting the surface area. With the ongoing reaction and the influence of KH550, the surface area was observed to further decrease, with the rate of decrease gradually weakening after 24 h. Overall, an initial increase in surface area was noticed at the start of the reaction, followed by a downward trend as the reaction time progressed. The rate of decrease in surface area slowly weakened after 24 h, indicating this as the likely optimal reaction time. At the same time, considering the impact of the modification time on morphology, this study determined 24 h to be the optimal modification time. The modified HAp used thereafter was obtained under this condition.

### 3.5. X-ray Photoelectron Spectroscopy (XPS) Analysis of Modified Tubular HAp

**Figure 6:**
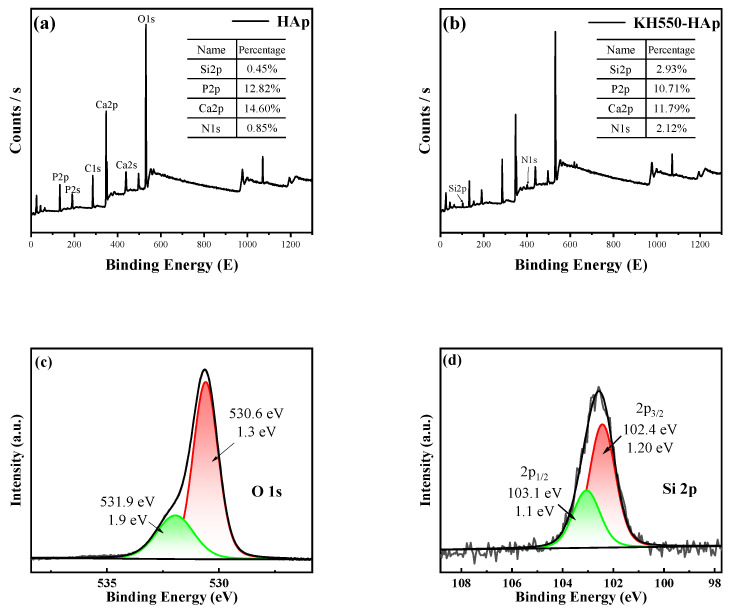
XPS images of HAp before (**a**) and after modification (**b**). (**c**,**d**): peak plots of O and Si.

X-ray photoelectron spectroscopy (XPS) was used to analyze HAp and KH550-HAp, as shown in Figure 6. Figure 6a depicts the total spectrum of HAp, and Figure 6b illustrates the total spectrum of KH550-HAp. In Figure 6a, six peaks can be observed, representing P 2p, P 2s, C 1s, Ca 2p, and O 1s. Compared with Figure 6a, Figure 6b also shows six peaks, in addition to two peaks at 102.58 eV and 399.43 eV, representing the Si 2p and N 1s structures on KH550-HAp, respectively, indicating that KH550 has successfully grafted onto the tubular HAp. Additionally, significant changes in the contents of the Si and N elements were observed following modification, as indicated by the comparison of atomic percentages, further confirming the successful grafting of KH550. In the XPS analysis, the photoelectron spectra in Figure 6c,d exhibit distinct peak features. These peaks, after precise fitting analysis, align well with the theoretically predicted characteristic peaks of the Si and O elements. This result unequivocally indicates the presence of Si and O on the surface of the material under study. Such a fitting analysis provides vital information about the surface chemical composition and electronic structure of the material, further confirming the distribution and state of the Si and O elements on the material’s surface. Moreover, the observed peak width ranging from 102.4 eV to 103.1 eV indicates that Si is present in the form of organosilanes in the HAp samples.

### 3.6. Mechanical Properties

**Figure 7:**
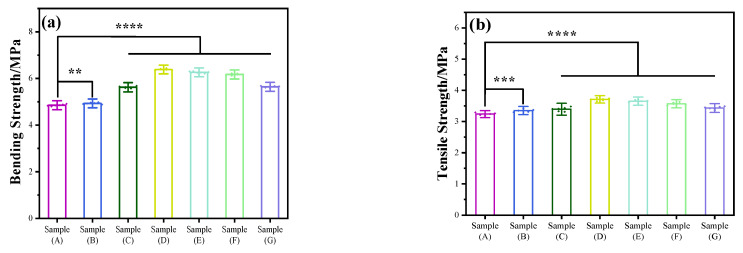
The bending and tensile strengths of the samples formed at different HAp contents. ((**a**): bending strength, (**b**): tensile strength). The samples (A–G) respectively represent the mass content percentage of HAp as: 0, 2, 4, 6, 8, 10, 15%. N = 5. **: *p* < 0.01, ***: *p* < 0.001 and ****: *p* < 0.0001.

As depicted in Figure 7, this study conducted tests on the tensile and bending strength of PCL/KH550-HAp using various formulations. In this experiment, Group A utilized pure PCL samples, which exhibited flexural strength and tensile strength measured at 4.85 ± 0.038 Mpa and 3.24 ± 0.038 Mpa, respectively. With the gradual incorporation of KH550-HAp, the experimental samples showed a significant enhancement in both their blending and tensile strength. Particularly in group D (containing 6% KH550-HAp), these properties peaked at 6.38 ± 0.037 Mpa and 3.71 ± 0.040 Mpa, respectively. Compared to the blank control group, there was an increase of 31.54% in flexural strength and 14.50% in tensile strength. When the addition amount of KH550-HAp exceeds 6%, the tensile strength and bending strength of this material decreased to a certain extent. It may be that the high aspect ratio of the tubular HAp material has a good reinforcing effect on the mechanical properties of the material. When the filler increased, the aggregation of KH550-HAp makes the prepared scaffold material uneven, which also affects the mechanical properties of the material. By comparing the tensile strength and bending strength, it can be found that the KH550-HAp filler improved the bending resistance more significantly. The main reason is that the KH550-HAp filler is in a nanowire shape. The toughness of this filler would play a role in resistance to external stress when the PCL/KH550-HAp scaffold material is subjected to external stress, especially when bending occurs.

### 3.7. Porosity

**Figure 8:**
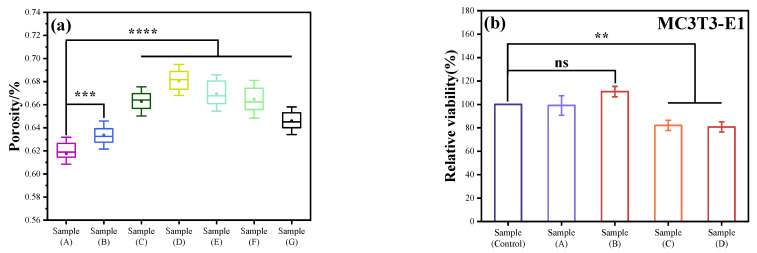
(**a**) The porosity of molding under different solid–liquid ratios. The samples (A–G) respectively represent the mass content percentage of HAp as: 0, 2, 4, 6, 8, 10, 15%. N = 9. (**b**) The relative cell survival rate of different components. N = 3. ns: Non-significant differences. **: *p* < 0.01, ***: *p* < 0.001, and ****: *p* < 0.0001.

The determination of porosity is illustrated in Figure 8a. As the concentration of HAp incrementally increases, there is an initial ascension in porosity, which subsequently diminishes, peaking at Sample D (68.04%). Mirroring the mechanical property outcomes, a minimal HAp concentration may lead to a slight surge in the composite material’s porosity. This can be attributed to the HAp particles potentially acting as nuclei, prompting the polymer to coalesce, forming porous structures. However, with further elevation in HAp content, the composite’s porosity commences its decline. The introduced HAp occupies some of the pre-existing voids, potentially leading to a more compact material configuration, resulting in a diminished porosity. Furthermore, the agglomerated HAp could lead to a non-uniform distribution of porosity within the material, consequently impacting its porosity characteristics.

Mechanical properties are quintessential in ensuring the stability and reliability of biological scaffolds within the organism. Furthermore, porosity directly affects cellular adhesion, proliferation, and tissue regeneration. Moreover, porosity profoundly influences the biodegradation rate of the scaffold and the formation of new tissues. Consequently, these two parameters are deemed pivotal criteria for evaluating the quality of scaffold materials. Through an intricate analysis of the material’s porosity and mechanical properties, the team discerned a significant deterioration in both properties when the inclusion of modified HAp surpassed 6%. In light of these findings, subsequent experiments solely focused on the parameters of Samples A through E.

### 3.8. Detection of Cytotoxicity Using the CCK8 Method

This study used the CCK8 method to detect the effect of samples on mouse osteoblasts. The experimental results are shown in Figure 8b. Sample A is a pure PCL resin processing material, with a relative cell survival rate of 99.0163%, which is basically the same as the control sample. Sample B is a PCL/HAp composite material, with a relative cell survival rate of 110.8207%, which is 10% higher than the pure resin material. This is because HAp promotes the growth of osteoblasts, and the effect is significant. Sample C is a PCL/KH550-HAp composite material, and the relative cell survival rate is only 81.98426%, which is 28.8% lower than Sample B, indicating that the existence of KH550 significantly inhibits the survival of MC3T3-E1 cells. Sample D is a PCL/drug-loaded KH550-HAp composite material, with a relative cell survival rate of 80.63519%. The difference between the cell viability and Sample C is not significant, indicating that a small number of drugs have little effect on the growth of MC3T3-E1 cells. However, in general, the cytotoxicity of cells was evaluated according to the toxicity grading method in the United States Pharmacopoeia. The evaluation criteria were: RGR ≥ 75%, cytotoxicity grade zero or one, and qualified [36,37]. Therefore, the conclusion can be drawn that the cell viability of the bone scaffold material prepared in this study meets the standard.

### 3.9. Surface Morphology of Sample Impact Section Analysis

**Figure 9:**
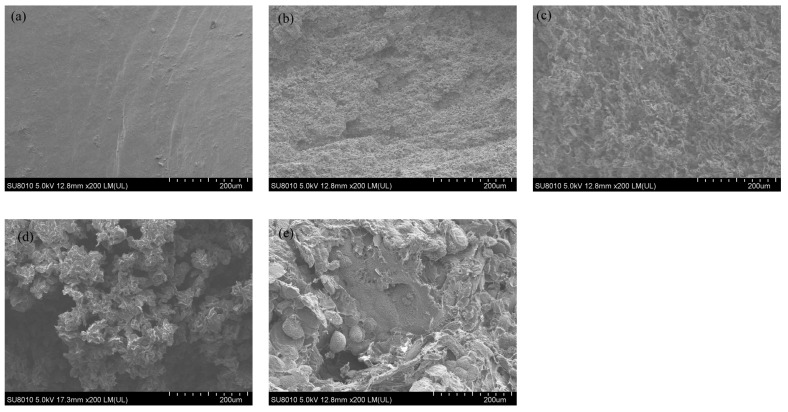
(**a**–**e**) represents the effect of different HAp quality ratio (0, 2, 4, 6, and 8) $w_{t}\%$ on the surface morphology of the scaffold material.

The present work aimed to investigate how the sectional morphology and structure of a material can impact its properties. To achieve this, a SEM was utilized to observe the PCL/KH550-HAp material with different HAp addition amounts. As illustrated in Figure 9, Sample A was devoid of HAp. In contrast, the HAp concentration was 2% in Sample B, 4% in Sample C, 6% in Sample D, and 8% in Sample E. From this figure, it can be clearly observed that with the gradual addition of HAp, the porosity of the material progressively increases, peaking in Sample D. Subsequently, it is evident that some exposed HAp began to agglomerate. This phenomenon mat be attributed to the inadequate encapsulation capability of PCL. The issues arising from this insufficiency further led to a noticeable decline in several properties of the materials in Sample E, such as porosity and mechanical strength. The agglomeration of HAp may result in stress concentration when the material is subjected to forces, potentially one of the reasons for the decreased mechanical performance in Sample E.

Therefore, the KH550-HAp/PCL material in Sample D (6% of HAp-modified content) is the best formula for the experiment, and the scaffold material prepared under this formula will be used in subsequent experiments.

### 3.10. Drug-Release Capability

**Figure 10:**
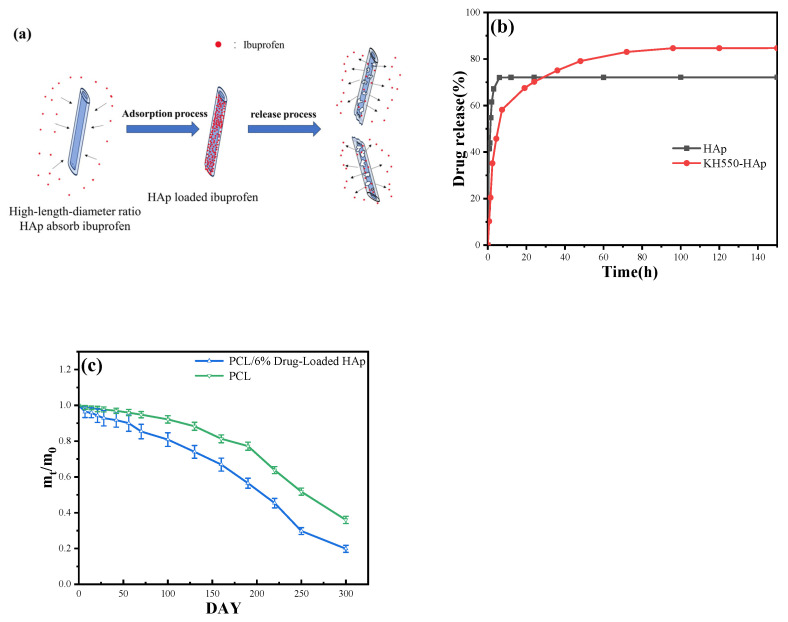
(**a**) The mechanism of drug release. (**b**) Drug release–time curve of HAp and KH550-HAp. (**c**) Degradation of pure PCL and PCL/6% drug-loaded HAp in a pH = 7.4; PBS solution; n = 5.

The degradability of HAp varies under different pH conditions. In acidic environments, such as those typically found at inflammatory sites, HAp is more prone to dissolution, thereby facilitating drug release. Additionally, HAp can gradually degrade through cellular processes, such as absorption by osteoblasts. As HAp degrades, the adsorbed drugs are released, achieving a gradual drug release. The aforementioned process is illustrated in Figure 10a. At a regulated temperature of 36.5 °C and in a simulated drug-release environment with a PBS buffer solution at pH 7.4, Figure 10b compellingly illustrates that in contrast to the unmodified tubular HAp, which only maintained a release cycle of three hours, the KH550-modified HAp accomplished a prolonged release duration of 24 h, markedly enhancing its drug utilization efficiency. It may be due to the active properties of KH550 forming chemical bonds with the drug or the short-chain KH550 being absorbed by HAp, which forms a certain coating for drug loading and release. This process greatly improves the effect of drug release. The drug-release rate has achieved 85%, conspicuously surpassing the unmodified HAp. This underscores the significant enhancement in the drug delivery capabilities of the modified HAp.

### 3.11. In Vitro Degradation Analysis of Scaffold Materials

This study aimed to investigate the in vitro degradation of two materials: PCL/6% drug-loaded HAp, and pure PCL resin. Both sets of materials were placed in a PBS buffer solution, simulating a physiological environment with a pH of 7.4. The degradation process was measured by tracking the mass of the materials at different time points. As shown in Figure 10c, in the first 10 days, the mass decline rates of PCL/6% drug-loaded HAp were significantly higher than that of pure PCL resin. The difference in amplitude was almost equivalent to the drug release. The rapid decrease in mass may be attributed to the drug being released from the drug-loaded HAp. After 25 days, the degradation rate of pure PCL resin was found to be lower than that of PCL/6% drug-loaded HAp. The composite material exhibited a notable alteration in its degradation rate between 220 and 250 days, whereas pure PCL manifested a significant shift at 190 days. After 250 days, the degradation rates of all the materials decelerated. By the 300^th^ day, approximately 80.19% of the composite material had degraded, in contrast to 64.01% for pure PCL. It is possible that the surface of the inorganic material HAp contains numerous hydroxyl groups. These groups have a strong interaction with the particles in the PBS simulation solution and can enhance the material’s ability to mineralize biologically. This, in turn, can transform the preparation of PCL resin containing HAp from an organic compound to an inorganic one, leading to an improvement in the material’s degradation rate. When the content of HAp increases, the decomposition rate of the material is faster, indicating that the addition of HAp effectively enhances the mineralization ability of the composite.

### 3.12. Drug-Release Kinetics of Modified HAp

**Figure 11:**
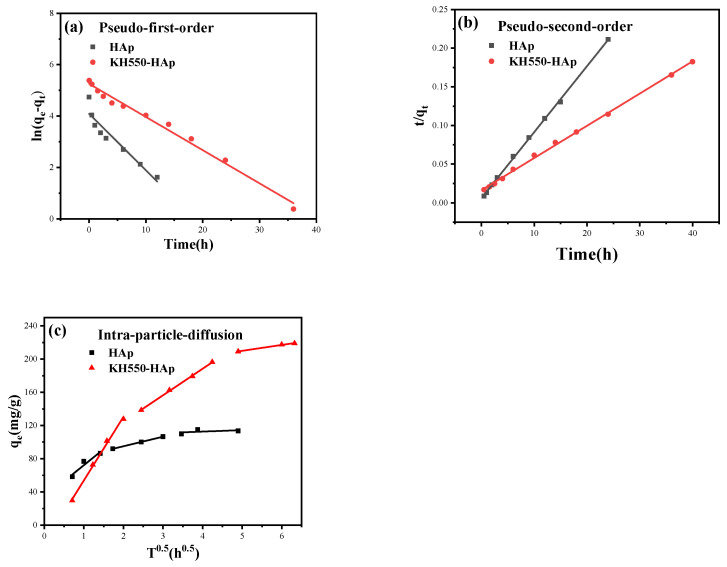
(**a**) Pseudo-first-order kinetic curve. (**b**) Pseudo-second-order kinetic curve. (**c**) Intra-particle diffusion.

The kinetic parameters related to these models are tabulated in Table 2. The pseudo-first- and second-order kinetics of modified HAp and pre-modified HAp are shown in Figure 11a,b, respectively. Upon analyzing the kinetic models both before and after modification, the equilibrium adsorption capacities under the pseudo-first-order kinetic model are discerned to be 59.7160 mg/g and 191.8761 mg/g, respectively. This represents a marked enhancement of 221.31% post-modification. Under the pseudo-second-order kinetic model, the maximum adsorption capacities are ascertained to be 239.8082 mg/g and 117.0960 mg/g, respectively, reflecting a post-modification increase of 104.80%. A comparison reveals that the R-value for the pseudo-second-order kinetic model aligns more congruently with one than that of the pseudo-first-order model. Moreover, the maximum adsorption capacity ascertained from the pseudo-second-order kinetic model resonates more accurately with the experimental data than its first-order counterpart. Consequently, it can be deduced that the adsorption process of the IBU drug on tubular HAp, both pre- and post-modification, predominantly conforms to the pseudo-second-order kinetic model, with chemical adsorption holding paramount significance. Further probing into the diffusion mechanisms pre- and post-modification using the particle diffusion model reveals, as evident from Figure 11c, that the initial curve post-modification is steeper, implying a superior adsorption process of IBU from the solution surface of the modified tubular HAp compared to its unmodified counterpart. This tangentially indicates an enhanced adsorption capacity post-modification. The subsequent curve represents the internal diffusion process where HAp manifests weaker diffusion, whereas post-modification, this internal diffusion is markedly pronounced. The final segment of the curve signifies the attainment of adsorption equilibrium. In summation, both membrane diffusion and internal particle diffusion concurrently transpire throughout the entire adsorption experiment, with a conspicuous increase in adsorption capacity and more pronounced internal particle diffusion post-modification.

### 3.13. Cytotoxicity Test

**Figure 12:**
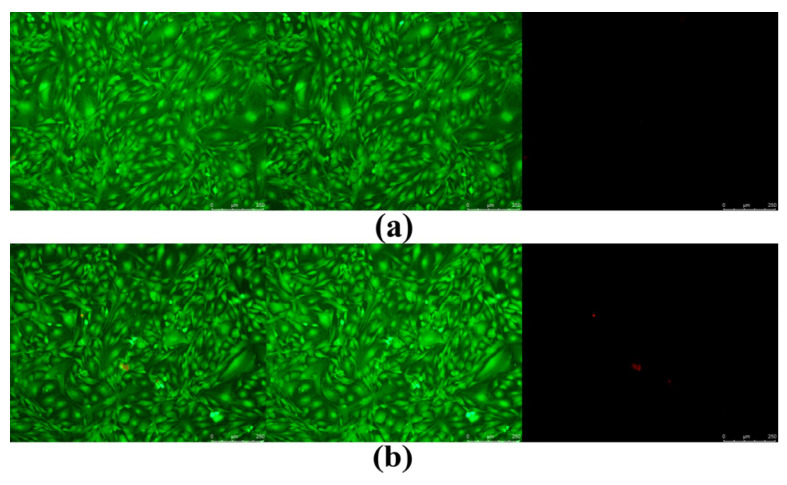
Fluorescence staining of live–dead cells with different components. (**a**) Pure PCL resin. (**b**) PCL/drug-loaded HAp composite.

To explore the cytotoxicity of scaffold materials (6% drug-loaded HAp contained), the effect of the samples on the activity of mouse osteoblasts (MC3T3-E1 cells) was detected using the live–dead staining method. As shown in Figure 12, the first column of the figures represents the overlay of the second and third columns, whereas the second and third column figures exhibit the fluorescent signals of the living and dead cells, respectively. Living cells exhibit green fluorescence, while dead or damaged cells display red fluorescence. The green fluorescent images of living cells in two sample groups were prominent, while the fluorescent images of dead cells, represented in red, were barely visible. This suggests that the scaffold materials utilized were supportive of regular cell growth and implies that the cytotoxicity of the two sample groups was negligible. Both the CCK8 method and the live–dead staining method arrived at the same conclusion regarding the results of sample detection.

### 3.14. Future Direction of KH550-HAp/PCL

The HAp/PCL material has been proven to possess good biocompatibility [38,39]. Composite materials with higher levels of porosity were obtained after the modification with KH550. This provides a possibility for loading BMP proteins to induce autogenous bone regeneration [40]. Furthermore, the degradation of the implanted bone materials can provide space for new bone regeneration. Therefore, it has a very broad application prospect in clinical practice. Moreover, this research offers a new perspective on the selection of artificial scaffold materials [41,42].

## 4. Conclusions

In this study, a composite material based on PCL with modified HAp with a high aspect ratio as an additive was prepared. When the addition of HAp reached 6%, the material exhibited good mechanical properties and porosity. Under a SEM, a three-dimensional loose porous structure was observed. This not only facilitates the excretion of cellular metabolic waste but also accelerates the degradation of the composite material, providing space for the regeneration of new bone cells.

## Figures and Tables

**Table 1:** The contents of HAp across different groups (forming volume: 10 mL).

Sample	A	B	C	D	E	F	G
Powder (g)	3	3	3	3	3	3	3
HAp	0%	2%	4%	6%	8%	10%	15%

**Table 2:** The adsorption kinetic model parameters of HAp and KH550-HAp.

Kinetic Model	Parameter	HAp	KH550-HAp
	K_1_ (min^−1^)	0.2199	0.12908
Pseudo-first-order	q_e_ (mg/g)	59.7160	191.8761
	R	0.9508	0.9937
	K_2_ (g/mg·h)	0.01220	0.001076
Pseudo-second-order	q_e_ (mg/g)	117.0960	239.8082
	R	0.9995	0.9995
	K_ip-1_ (mg/g·h^−0.5^)	38.6075	76.6329
	C (mg/g)	33.6565	−22.7467
	R	0.9619	0.9979
	K_ip-2_ (mg/g·h^−0.5^)	11.5269	31.9712
Intra-particle diffusion	C (mg/g)	71.9607	60.5780
	R	0.9999	0.9995
	K_ip-3_ (mg/g·h^−0.5^)	3.0360	7.0771
	C (mg/g)	105.6376	174.6759
	R	0.5009	0.9967

## Data Availability

Data are contained within the article.
